# Supplementary material for: Splice-Junction-Based Mapping of Alternative Isoforms in the Human Proteome
Source: Cell Rep. Author manuscript; Available in PMC 2020 Jan 15. (PMC6961840; doi:10.1016/j.celrep.2019.11.026)
Supplement: 3 [file NIHMS1546469-supplement-3.zip › DF2/PXD009737/TestisTrypsin-146-A5PLN9-FYNAETDEVFLEAQIQNMTTSPMFMEK.pdf]

A

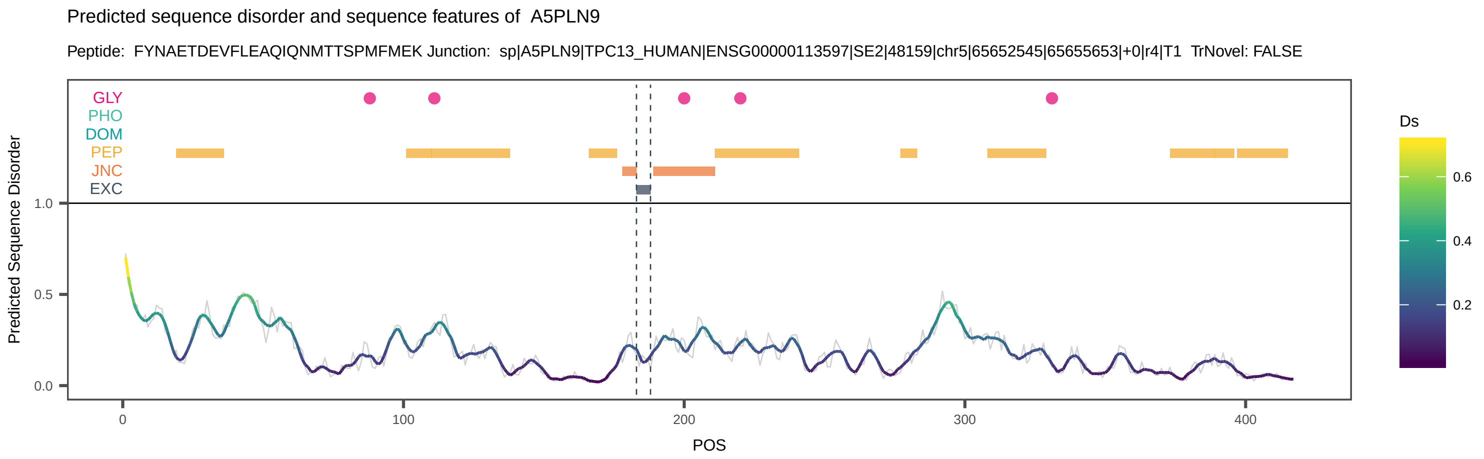

Predicted sequence disorder and sequence features of A5PLN9
Peptide: FYNAETDEVFLEAQIQNMTTSPMFMEK Junction: sp|A5PLN9|TPC13_HUMAN|ENSG00000113597|SE2|48159|chr5|65652545|65655653|+0|r4|T1 TrNovel: FALSE
GLY
PHO
DOM
PEP
JNC
EXC
Predicted Sequence Disorder
1.0
0.5
0.0
0
100
200
300
400
POS
Ds
0.6
0.4
0.2

B

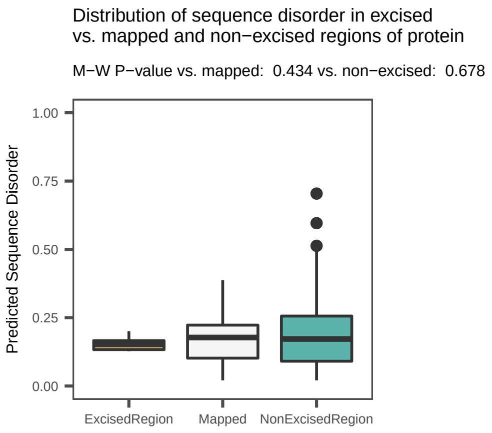

Distribution of sequence disorder in excised
vs. mapped and non−excised regions of protein
M−W P−value vs. mapped: 0.434 vs. non−excised: 0.678
Predicted Sequence Disorder
1.00
0.75
0.50
0.25
0.00
ExcisedRegion
Mapped
NonExcisedRegion

C
